# Exercise in Sub-zero Temperatures and Airway Health: Implications for Athletes With Special Focus on Heat-and-Moisture-Exchanging Breathing Devices

**DOI:** 10.3389/fspor.2020.00034

**Published:** 2020-04-28

**Authors:** Helen G. Hanstock, Mats Ainegren, Nikolai Stenfors

**Affiliations:** ^1^Swedish Winter Sports Research Centre, Department of Health Sciences, Mid Sweden University, Östersund, Sweden; ^2^Sports Tech Research Centre, Department of Quality Management and Mechanical Engineering, Mid Sweden University, Östersund, Sweden; ^3^Department of Public Health and Clinical Medicine, Umeå University, Umeå, Sweden

**Keywords:** asthma, airway inflammation, exercise, exercise-induced bronchoconstriction (EIB), cross-country skiing, winter sports

## Abstract

Asthma is highly prevalent among winter endurance athletes. This “occupational disease” of cross-country skiers, among others, was acknowledged during the 1990s, with the pathogenesis attributed to repeated and prolonged exposure to cold, dry air combined with high rates of ventilation during exercise. Nevertheless, more than 25 years later, the prevalence of asthma among Scandinavian cross-country skiers is unchanged, and prevention remains a primary concern for sports physicians. Heat-and-moisture-exchanging breathing devices (HMEs) prevent exercise-induced bronchoconstriction in subjects with pre-existing disease and may have potential as a preventative intervention for healthy athletes undertaking training and competition in winter endurance sports. Herein we firstly provide an overview of the influence of temperature and humidity on airway health and the implications for athletes training and competing in sub-zero temperatures. We thereafter describe the properties and effects of HMEs, identify gaps in current understanding, and suggest avenues for future research.

## Introduction

Athletes training and competing outdoors will, most likely, be periodically exposed to cold, and sometimes sub-zero air. In fact, around 4 million people live in the Arctic region (Larsen and Fondahl, 2015) and many more are intermittently exposed to sub-zero temperatures during work and leisure. Exposure to cold air is associated with increased morbidity and mortality in the general population (Rocklöv and Forsberg, 2008). Possible explanations for these observations include increased bacterial survival leading to more airway infections (Handley and Webster, 1995), chilling of the nasal epithelium leading to inhibition of respiratory defense against pathogens (Eccles, 2002), cutaneous vasoconstriction leading to increased blood pressure and cardiac load (Cheng and Su, 2010), increased coagulation (Hampel et al., 2010) and bronchoconstriction leading to airway obstruction (Koskela and Tukiainen, 1995).

Studies from Finland show that up to 50% of the population report at least some cold-related symptoms (Harju et al., 2010; Näyhä et al., 2011) and that symptoms appear to be more common in women than men (Näyhä et al., 2011). A recent study found that experimental exposure to sub-zero temperatures at rest and when performing light exercise elicited 50 distinct symptoms among healthy subjects and patients with obstructive lung disease (Sjöström et al., 2019). Respiratory symptoms are also very common among children undertaking physical activity in cold temperatures (Rasi et al., 2017). Potentially for these reasons, up to one third of asthmatic individuals report avoidance of outdoor activities during cold spells (Millqvist et al., 1987). Thus, cold climates can present a challenge to facilitate physical activity from a public health perspective, and moreover individuals who habitually undertake physical activity in cold environments may experience airway symptoms and/or morbidity as a result of their training.

Winter endurance athletes, such as cross-country skiers, frequently undertake prolonged exercise in cold environments and report an increased prevalence of airway symptoms, bronchial hyper-reactivity and asthma (Carlsen et al., 2008). Asthma is a heterogeneous chronic inflammatory disease of the airways, characterized by recurrent episodes of bronchial constriction and airflow limitation that presents with symptoms such as cough, wheezing, and breathlessness. In Sweden, the prevalence of asthma among adolescents and young adults aged 16–24 years is around 9% (Wennergren et al., 2010). In 1994, 15% of 299 Swedish athletes from upper secondary school cross-country ski teams and the Swedish army reported physician-diagnosed asthma (Larsson et al., 1994). More recently, asthma prevalence among Swedish elite cross-country skiers has been estimated at 29–35%, with onset typically occurring during adolescence (Norqvist et al., 2015; Eriksson et al., 2018).

Two major mechanisms may explain the increased prevalence of exercise-induced asthma in winter endurance athletes. The airways condition inspired air to 37°C and 100% relative humidity (equivalent to 44 mg·L^−1^ H_2_O) which leads to evaporative water loss from the airway surface (Kippelen et al., 2018). This evaporative water loss cools the mucosa, leading to vasoconstriction, reactive hyperemia, vascular leakage, and edema. Meanwhile, dehydration of the airway mucosa increases osmolarity of the periciliary fluid and stimulates the release of mediators that trigger smooth muscle contraction (Anderson and Daviskas, 2000; Anderson and Kippelen, 2008). The physiological stimuli of mucosal cooling and dehydration are likely to be exacerbated by low temperatures, dry climates, and prolonged, high rates of ventilation, each hallmarks of the training environment for winter endurance sports. Heat-and-moisture-exchanging breathing devices (HMEs), discussed in detail in the latter part of this article, may thus directly intervene with the proposed pathway of airway injury among winter endurance athletes.

The aim of this narrative review is therefore twofold; to first provide an overview of pathophysiological responses to exercise in sub-zero temperatures, and secondly, to review the potential of HMEs to prevent airway pathophysiological responses to cold air exercise. Throughout we maintain a focus on the implications for athletes and practitioners undertaking training and competition in these environments and highlight gaps in current knowledge with potential for translation into practical recommendations.

## Pathophysiology of Exercise-Induced Asthma In Cold Weather Athletes

### Conditioning of Inspired Air

The nasal passage is an important structure which serves to condition inspired air to near-alveolar conditions. Nasal breathing appears to defend against airway cooling when inhaling sub-zero air (Griffin et al., 1982). Indeed, anthropologists have noted longer, narrower, nasal cavities in human populations native to cold, dry climates, compared to humans from hot and humid climates, suggesting an important role of the nasal cavity in warming and humidifying cold, dry air (Noback et al., 2011). While nasal breathing is the norm in healthy individuals at rest, during exercise breathing patterns habitually switch to oronasal breathing at minute ventilation (${\overset{´}{V}}_{\text{E}}$) rates around 35 L·min^−1^ (Niinimaa et al., 1980). Oral breathing appears to be less efficient at conditioning inspired air, leading to lower inspired air temperatures (Griffin et al., 1982) and drying of the upper airway mucosa (Verma et al., 2006). A few individuals are able to maintain predominantly nasal breathing patterns even during high-intensity exercise and it would be interesting to investigate whether habitual breathing during exercise is associated with development of airway hyper-responsiveness. However, for the majority of individuals exercising at high to maximal exercise intensities, oronasal breathing predominates, which could in turn impair the ability of the airways to condition inspired air.

Thermal mapping of the airways indicates that room temperature air (26°C, 8.8 mg·L^−1^ H_2_O) is warmed during tidal breathing to ~32°C at the tracheal carina and 35.5°C in the subsegmental bronchi. At a ${\overset{´}{V}}_{\text{E}}$ of 100 L·min^−1^, inspired air temperatures fall by ~2°C at each location. Meanwhile, inhaled air at −18°C and 0 mg·L^−1^ H_2_O reaches only 23°C at the carina and 31°C at the bronchial level (McFadden et al., 1985). Light to moderate-intensity exercise in −20°C elicits exhaled air at around 28–31°C and relative humidity >90% (Cain et al., 1990). Thus, it is reasonable to suggest that below certain sub-zero temperatures, the airways are unable to fully warm and humidify inspired air. The challenge to the airways to condition inspired air is further amplified at ventilatory rates typically attained during moderate to high-intensity exercise and exacerbated by oronasal breathing.

### Quantification of Temperature and Humidity in Inspired Air

**Figure 1:**
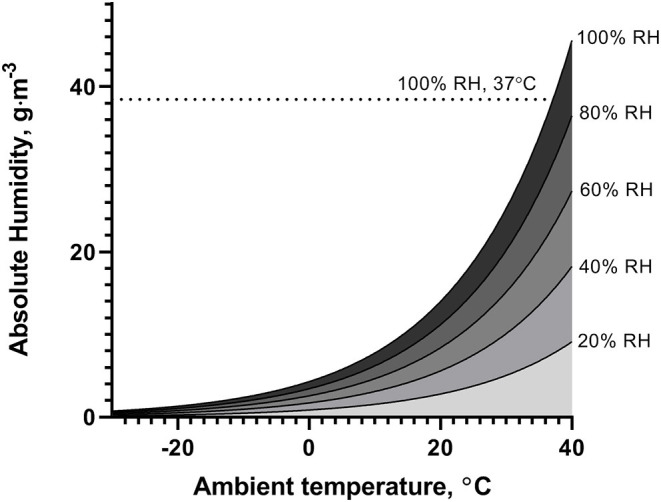
Illustration of absolute humidity of ambient air at temperatures between −30 and 40°C, for different levels of relative humidity (RH).

Decreases in environmental humidity and temperature present an additional challenge to the airways to adequately condition inspired air. During hyperpnea, which naturally occurs during high-intensity exercise, heat and water losses further increase, especially in the upper airways, causing airway dehydration and inducing a hypertonic periciliary fluid (Daviskas et al., 1991). Moreover, cold air holds less water vapor, so is dry by default compared to temperate air and may exacerbate airway dehydration. Magnus' exact formula (Rindert, 1993) may be used to derive the absolute humidity for a given temperature, relative humidity, and pressure. Figure 1 illustrates the challenge for the airways to heat and humidify inhaled air to near core temperature and saturated with water vapor (absolute humidity = 38.5 g/m^3^ at 37°C and 100% RH), at different ambient temperatures and levels of relative humidity (assuming ambient pressure = 1,013 hPa).

### Effects of Whole-Body Exposure to Cold Air

Individuals participating in winter endurance sports not only inhale cold, dry air but experience whole-body exposure to sub-zero environments during training and competition. Controlled environment conditions generated by environmental chambers permit simulation of the training environment and experimental investigation of systemic physiological responses to exercise in cold climates. To date, exposure studies have frequently focused on lung function and elucidating mechanisms, often using isolated cold and/or dry air inhalation as an experimental stimulus (Strauss et al., 1977; Deal et al., 1979; Eiken et al., 1989). However, airway obstruction has been shown to be more pronounced when the face (Koskela and Tukiainen, 1995; Josenhans et al., 2011) or nose (Fontanari et al., 1996, 1997) are exposed to cold air among both healthy and asthmatic subjects. This indicates that whole-body exposure, as opposed to isolated hyperpnea of cold, dry air, may be a preferred approach to evaluate airway effects of cold air exposure as well as systemic physiological responses to cold. Furthermore, little distinction in the literature has been made between “cold” air of different temperatures, with studies investigating airway responses to cold air spanning a 50°C temperature range (10 to −40°C) (Cain et al., 1990; Eschenbacher et al., 1992). With winter sports in mind, we herein focus on studies that have investigated airway and physiological effects of whole-body exposure to sub-zero air within typical temperature ranges experienced by winter endurance athletes; that is, 0°C to around −20°C.

### Effects of Cold Air on Lung Function

It has been shown that even 5–10 min of whole-body *resting* exposure to −17°C may trigger acute decreases in forced expiratory volume in 1 s (FEV1), the classical measurement of airway obstruction, in both healthy subjects and patients with obstructive lung disease (asthma or chronic obstructive pulmonary disease (COPD) (Koskela and Tukiainen, 1995; Koskela et al., 1996). It has then been consistently observed that exercising in sub-zero temperatures induces bronchial obstruction in subjects with respiratory disease (Koskela et al., 1994, 1998; Stensrud et al., 2007). When *healthy* subjects perform physical activity to exhaustion, acute bronchial obstruction has also been detected at sub-zero temperatures (Therminarias et al., 1998; Kennedy and Faulhaber, 2018), but the results are inconsistent, with lower-intensity protocols revealing no effect on pulmonary function (Pekkarinen et al., 1989; Chapman et al., 1990). Discrepancies in the current literature may be explained not only by heterogeneity in exercise intensity, duration and modality but potentially also by differences in temperature and relative humidity across a relatively small number of studies (Table 1).

**Table 1:** Experimental studies that have employed environmental chamber models to examine acute effects of short-term, whole-body exposure to sub-zero temperatures on lung function and other physiological variables.

**References**	**Subjects**	**Temperature (^**°**^C)**	**Humidity**	**Duration (minutes)**	**Exercise intensity**	**Outcome**
Pekkarinen et al. (1989)	Healthy males, *n* = 20	−20	~50% RH	8–17	70–75% of HR*_*max*_*	No effect on FVC, FEV_1_, PEF, MEF
Chapman et al. (1990)	Healthy, *n* = 12	−11	<2% RH	30	80% of $\overset{\cdot }{V}$O_2max_	No effect on pulmonary function indices
Koskela et al. (1994)	Asthma, *n* = 19	−20	Not reported	10	≥70% of HR*_*max*_*	FEV_1_ ↓
Koskela and Tukiainen (1995)	Healthy, *n* = 15, and asthma, *n* = 10	−17	Not reported	10	Rest	FEV_1_ ↓ both groups
Koskela et al. (1996)	COPD, *n* = 20, and healthy, *n* = 10	−17	<1.75 mg/L	5–10	Rest	FEV_1_ ↓ both groups
Koskela et al. (1998)	COPD, *n* = 14	−19	Not reported	10	Until exhaustion	FEV_1_ ↓ Exercise duration ↓
Therminarias et al. (1998)	Healthy well-trained males, *n* = 8	−10	Not reported	30	Until exhaustion	FEV_1_ and FEF75 ↓
Stensrud et al. (2007)	Exercise-induced asthma, *n* = 20	−18	39% RH	8	≥95% of HR*_*max*_*	$\overset{\cdot }{V}O_{2peak}$ running speed, ${\text{FEV}}_{1}^{\cdot }↓$
Kennedy and Faulhaber (2018)	Healthy females, *n* = 17	0 to −20	40% RH	28	Until exhaustion	FEV_1_ ↓

*FEF75, Forced expiratory flow at 75% forced vital capacity; FEV_1_, Forced expiratory volume in 1 s; FVC, Forced vital capacity; HR_max_, Maximal heart rate; MEF, Maximal expiratory flow; PEF, Peak expiratory flow; $\overset{\cdot }{V}$O_2peak_, Peak oxygen uptake; $\overset{\cdot }{V}$O_2max_, Maximal oxygen uptake*.

Among non-asthmatic skiers, up to 75% have returned positive responses to methacholine challenge (Karjalainen et al., 2000). Furthermore, a higher rate of positive methacholine challenge arose during winter in competitive speed skaters (Kurowski et al., 2018a) and methacholine reactivity was increased after the coldest period of the year in cross-country skiers (Heir and Larsen, 1995). However, some of the hallmarks of airway inflammation observed in cross-country skiers are not consistent with those of atopic asthma, eosinophilic asthma or traditional models of exercise-induced bronchoconstriction, in line with the suggestion that “sports asthma,” including that exacerbated by training in cold and dry air, has a distinct phenotype (Couto et al., 2015). Potentially for this reason, classical screening tests such as EVH have been shown to have poor agreement with a field test for exercise-induced bronchoconstriction (EIB) induced by exercise in cold environments, and it has been suggested that more research may be required to explore the best types of screening test for EIB in cold weather athletes (Kennedy et al., 2019).

### Effects of Cold, Dry Air on Airway Inflammatory Responses

A handful of studies have shown that elite cross-country skiers, including those with and without asthma, display markers of chronic airway inflammation and damage to the airway epithelial lining. These studies report increased bronchoalveolar and/or mucosal infiltration of eosinophils, neutrophils, macrophages, mast cells, and lymphoid aggregates in athletes training in cold climates compared to healthy controls, but fewer eosinophils and mast cells and more neutrophils than in subjects with asthma (Sue-Chu et al., 1998, 1999; Karjalainen et al., 2000). A more recent longitudinal study of female cross-country skiers over the course of a training season reported an increase in sputum eosinophils and lymphocytes between the beginning of the training year (late spring) and the peak of the winter competitive ski season (Kennedy et al., 2016). A mixed cohort of speed skaters and swimmers also presented with a similar inflammatory cytokine profile in exhaled breath condensate as that seen in asthmatics (Kurowski et al., 2018b). Furthermore, baseline TNF-α in exhaled breath condensate was positively correlated with percentage decreases in FEV_1_ following exercise challenge for both athletes during a hard training period and asthmatic subjects (Kurowski et al., 2018b). Among speed skaters reporting exercise-induced respiratory symptoms, IL-1RA was elevated during the winter training period compared to asymptomatic athletes (Kurowski et al., 2018a). Together these observations suggest a multifactorial inflammatory profile may develop during winter training in athletes that may be associated with symptoms and/or lung function.

However, very few studies have examined acute inflammatory responses to exercise in cold under standardized environmental conditions in either individuals with asthma or healthy subjects. Performing 2 h light exercise in −23°C has been shown to increase number of granulocytes and macrophages in the lower airways in healthy subjects (Larsson et al., 1998). However, as highlighted by Bonsignore and colleagues as long ago as 2003, there remains a gap in current knowledge about how acute exercise sessions performed in cold may acutely influence markers of inflammation local to the airways (Bonsignore et al., 2003). Systemic immune responses are typically not influenced by exercise in cold (Castellani et al., 2002), however, exercise duration and intensity are known to substantially influence immune responses to exercise, with prolonged, moderate-intensity exercise provoking greater *in vivo* immune perturbations than short, high-intensity exercise (Diment et al., 2015). On the other hand, it has been observed that bronchial hyper-reactivity to methacholine increases in association with volume of physical activity at higher exercise intensities (>90% maximal heart rate) (Heir and Larsen, 1995), and anecdotal reports from coaches and athletes have informed us that elite skiers tend to report respiratory symptoms more frequently after sprint competitions (typically 3–4 min duration at near-maximal intensities). Therefore, it would be pertinent to investigate whether inflammatory and immune markers local to the airways are acutely influenced by short, high-intensity exercise (i.e., near-maximal ${\overset{\cdot }{V}}_{\text{E}}$) or prolonged, moderate-intensity exercise (i.e., high area-under-curve for ${\overset{\cdot }{V}}_{\text{E}}$).

### Effects of Cold, Dry Air on Airway-Related Symptoms

Upper and lower respiratory symptoms are common among winter athletes (Svendsen et al., 2016; Valtonen et al., 2019) with as many as 80% reporting sporadic exercise-associated respiratory symptoms (Kurowski et al., 2018a). It has also been reported that occurrence of upper respiratory infections increases in the general population with cold temperature and low humidity (Mäkinen et al., 2009), which may partially explain increased symptom reports during winter. Although a proportion and perhaps indeed a majority of symptoms reported may be associated with viral upper respiratory tract infections (Spence et al., 2007; Valtonen et al., 2019), it is clear that not all symptoms are attributable to infectious causes (Rundell et al., 2001), particularly with regard to exercise-associated symptoms. For example, increased reporting of cough in cross-country skiers during the winter competitive season has been associated with sputum neutrophils and total yearly training (Kennedy et al., 2016), suggesting a direct localized association with both airway immune cells and training stress.

### Cold Effects on Performance

From a performance perspective, cross-country skiing double-poling performance has been shown to decline at −15 vs. 6°C whilst wearing a standard racing suit (Wiggen et al., 2016). Running time to exhaustion in cross country skiers has also been reported as shorter at −14°C than −4 and 1°C when wearing a standard cross-country racing suit (Sandsund et al., 2012). Considering both studies together, core temperature typically increases during exercise at −14 to −15°C but potentially to a lesser extent than at warmer temperatures (−9 to 20°C). Colder environments appear to produce greater reductions in skin temperature during exercise. Neither study found differences in $\overset{\cdot }{V}$O_2_ nor ${\overset{´}{V}}_{\text{E}}$ during maximal exercise tests across a broad range of environmental conditions (−15 to 20°C), but power output was lower during the first 8 min of the 20-min double poling test at −15°C compared to 6°C, suggesting that a reduction in skin temperature may lead to muscular cooling and reduced exercise economy (Wiggen et al., 2016). Muscular cooling may also be associated with increased risk of injury (Scott et al., 2016). Together these results suggest that in cross-country skiers without asthma, ventilatory rates and oxygen uptake are not significantly affected during high-intensity exercise at −15°C (Wiggen et al., 2016).

## Toward Temperature and Humidity Thresholds For Airway Pathophysiological Responses

Assuming that the increased risk of developing asthma among winter endurance athletes is due to repeated and prolonged inhalation of cold and dry air, a pertinent question arising from athletes, coaches and organizations in winter sports is whether specific ambient temperature and/or humidity thresholds can be defined below which airway damage is likely to occur during training or competition. From an epidemiological perspective, the threshold temperature for presentation of cold-related respiratory symptoms at a population-level is at sub-zero temperatures (−7 to −18°C) and slightly higher among subjects with lung disease (Harju et al., 2010).

The relative importance of inhaled air temperature vs. humidity on airway responses to the cold remains unclear. Low water content of inhaled air appears to be a stronger inducer of exercise-induced asthma than low temperature, at least at non-freezing temperatures (Hahn et al., 1984; Anderson et al., 1985; Eschenbacher et al., 1992). However, hyperpnea of sub-zero dry air has been shown to induce greater airway obstruction in asthmatic subjects compared to inhalation of dry air at room temperature (Zawadski et al., 1988), suggesting temperature itself factor that could exacerbate bronchoconstriction. It has even been reported that cold, damp air may elicit more symptoms in patients with asthma than cold, dry air (Millqvist et al., 1987). Taken together, there is a lack of agreement on critical thresholds for airway health risks, both in asthmatic and non-asthmatic subjects, with respect to inhaled air temperature and humidity; particularly for elite winter endurance athletes undertaking exercise in cold, dry conditions.

Anecdotally, it could be expected that training and competition temperatures in winter sports are frequently below 0°C but rarely below −20°C. Thus, perhaps the first important question for those participating in recreational activity and competitive sport in sub-zero climates is whether the challenge to the airways to humidify air varies substantially within this temperature range to increase risk of airway injury or presentation of respiratory symptoms. It is possible that it is not just the absolute humidity but the rate of cooling and/or condensation of expired air in sub-zero temperature that plays an important role in determining this.

### Temperature Limits for Competitions

The Federation Internationale de Ski (FIS) rules for international competition at the time of writing state that if the temperature is below −20°C at the coldest part of the course, a competition should be postponed or canceled. In the case of other challenging weather conditions such as strong winds or high humidity (as well as high temperature or heavy snowfall) the Jury may also decide to postpone or cancel the competition. For so-called “popular” competitions, FIS rules also recommend additional precautions, for example to provide recommendations regarding cold weather protection if the temperature is forecast to be between −15 and −25°C, and cancellation if the temperature in a major portion of the course is expected to be −25°C or below (Federation Internationale de Ski, 2018). It has been suggested via the FIS Medical Committee that temperature thresholds should be higher for long-distance races (>30 km, −16°C) than for shorter distances (<30 km, −18°C) and sprints (−20°C), and higher (−12°C) for children under 14 years (Lereim, 2007). Although such regulations may have been adopted by local or national organizations, these recommendations have not been implemented in international competitions to date. The International Biathlon Union (IBU) rules at the time of writing state that competitions should not be started if the air temperature is below −20°C at the coldest part of the site, but that if it is colder than −15°C then wind chill and humidity must be considered (International Biathlon Union, 1998).

### Are Current Temperature Limits Appropriate?

Most winter endurance sport governing bodies recommend lower temperature limits for competition. However, despite earlier commentaries on the potential respiratory health risks of undertaking competitions in very cold and dry climates (Kippelen et al., 2012; Sue-Chu, 2012), few explicitly cite concerns regarding airway health as a major rationale. Collectively, rules stipulated by governing bodies in winter endurance sports suggest little consensus in “safe” temperatures for competition for elite or amateur athletes.

It is clear that temperatures above the present limits stipulated by governing bodies may induce bronchoconstriction in subjects with asthma. Protection of the airways under such conditions would therefore seem warranted and it is notable that heat-moisture exchanging breathing devices are not prohibited in competition. Of further interest is the question about whether current competition temperature thresholds are safe for healthy athletes without asthma. During exercise at −23°C, cellular responses associated with airway inflammation have been observed in healthy individuals (Larsson et al., 1998). Airway inflammatory responses as well as epithelial injury have potential to underpin the development of asthma in at-risk individuals, but the existence of sub-clinical airway inflammation arising from exercise in cold in individuals without asthma is not well-defined, let alone threshold temperatures at which such responses could occur. Given development of asthma among winter endurance athletes typically presents during adolescence and later than in reference populations (Eriksson et al., 2018), there is also a need to investigate whether adolescents and young athletes are in need of more conservative guidelines to reduce risk of airway injury.

### Heat-and-Moisture-Exchanging Breathing Devices

**Figure 2:**
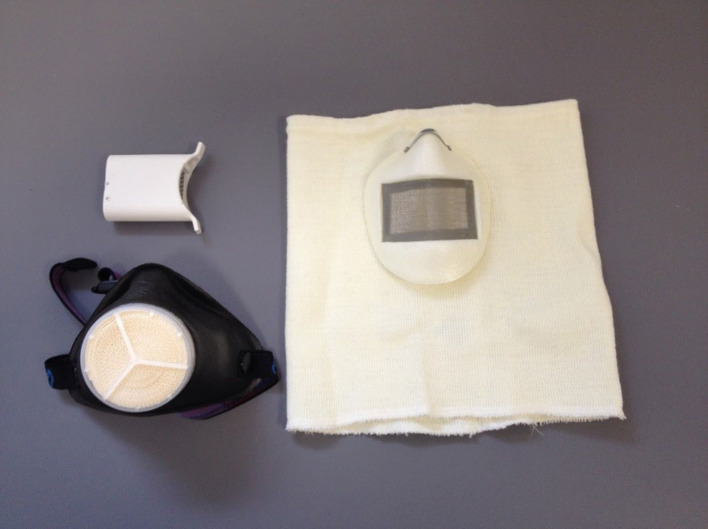
Three representative types of HME: Lungplus (upper left), AirTrim (lower left), and Jonaset 0602 (right).

Heat and moisture exchangers (HMEs) are simple and generally inexpensive tools that may offer protection to the airways from the potentially damaging effects of heavy exertion in sub-zero conditions. On the market there are essentially three different types of HME design, all with the common feature of a filter where exchange of inspiratory and expiratory air can take place. A representative selection of the three designs of HME is shown in Figure 2.

The first design of HME is held firmly with the mouth (Lungplus Info AB, Hörby, Sweden) and does not cover the nose, which then will maintain its usual function as heat exchanger and humidifier of inspired air. The second design of HME consists of a mask (Air Trim, Vapro Produktutveckling AB, Västerås, Sweden) covering the skin surface around both nose and mouth. This type of HME is held in place against the skin by means of a strap which is tensioned around the back of the head. The third type of HME has the filter fixed in a tube of some type of textile that is threaded over the head and covers most of the face including nose, mouth and neckline (Jonaset 0602, Suojalaite OY, Helsinki, Finland). The principle of a HME is that its inner surfaces and filters are heated and moistened by the exhaled air. The filter also constitutes a barrier that prevents the mixing of residual exhaled air with ambient air so that the volume and the surface inside the HME is prevented from being cooled and dehumidified with ambient air during the short time between exhalation and inhalation. Upon inhalation, cold and dry ambient air will therefore be able to be heated and moistened both by the filter and the remaining exhaled volume inside the HME. Differences in HME filter area, mesh density, and remaining expiratory volume should lead to differences in the ability to warm and humidify inhaled air. Some degree of heat impact from the friction between gas molecules and the filter is also conceivable but probably relatively small in context. The intended functionality of an HME is thus to provide a pre-station where cold and dry ambient air is partially warmed and humidified before inspiration and thus before the cold air reaches the upper airways.

### Influence of HME on Breathing and Inspired Air Composition

The use of an HME may have both positive and negative consequences for the user which may be affected by both the type of HME and intensity of activity. HME filters are effective at warming air as it passes through the filter, especially when the source air is cold (Nisar et al., 1992). However, the remaining volume of exhaled air inside the HME constitutes an effective increase in dead space leading to a decrease and increase in inspiratory O_2_ and CO_2_ fractions, respectively (Campbell et al., 2000). Also, breathing through the filter may to some extent increase resistance to breathing. The volume of increased dead space varies with design and the manufacturer of HME, like probably the resistance to breathing, and the affected inspiratory gas fractions can be compensated for by the fact that the tidal volume is increased correspondingly to the increase in HME dead space. However, at high ventilations, such as the rates attained during high-intensity cross-country skiing, it may be difficult to increase the tidal volume further. Also, increased ventilation confers an increased energy cost for the respiratory muscles to further overcome the elasticity of the lung tissues and to drive the flow through the HME filter. However, since cold and dry air can cause bronchoconstriction, which leads to negative consequences such as increased airway obstruction, hypoventilation, and altered alveolar gas fractions, it would be interesting to investigate whether the sum of resistance from airways plus HME would be less than without using HME. Thus, prevention of bronchoconstriction with an HME would be positive for ventilation, pulmonary gas exchange, and energy cost of breathing.

### HMEs Prevent Exercise-Induced Asthma

A handful of studies have demonstrated that HMEs can attenuate exercise-induced bronchoconstriction triggered by cold and/or dry air (Table 2). Despite heterogeneity in ambient/inspired air temperatures (23 to −25°C), humidity, exercise protocol intensity and duration, these observations suggest that use of HMEs may be an effective strategy for prevention of exercise-induced airway obstruction in patients with asthma exercising in cold and/or dry conditions. A common trait of these studies is that all used participants with asthma, with reductions in FEV_1_ as the primary endpoint. Three studies reported protective effects of HMEs to attenuate reductions in FEV_1_ after exercise in sub-zero climates (Nisar et al., 1992; Millqvist et al., 1995; Beuther and Martin, 2006), cool temperatures (Jackson et al., 2018) and dry, ambient temperatures (Brenner et al., 1980; Millqvist et al., 1995). In addition, breathing through unidirectional inspiratory and expiratory filters in a placebo mask (Millqvist et al., 1995), and use of a placebo filter (Beuther and Martin, 2006) did not attenuate bronchoconstriction compared to a no-mask condition, giving weight to the mechanism of heat-moisture exchange in the filter as the mode of protection imparted by the mask. It has been shown that the attenuation of bronchoconstriction achieved by use of an HME is comparable in magnitude to the protective effects imparted by pre-treatment with short-acting beta-2 agonists prior to exercise, and that combining the two strategies can fully prevent reductions in FEV_1_ (Millqvist et al., 2000).

**Table 2:** Studies showing that HMEs attenuate exercise-induced bronchoconstriction.

**References**	**Temperature (^**°**^C)**	**Humidity**	**Duration (minutes)**	**Exercise intensity/** **ventilation rate**	**Main finding**
Beuther and Martin (2006)	−15 to −25 Isolated breathing of sub-zero air	Not reported	10	85% $\overset{\cdot }{V}$O_2max_	Placebo: ΔFEV_1_ = −19 ± 4.9% HME: ΔFEV_1_ = −4.3 ± 1.6% fall in FEV_1_
Nisar et al. (1992)	−13 Isolated breathing of sub-zero air	Not reported	6	75% $\overset{\cdot }{V}$O_2max_	No HME: ΔFEV_1_ = −22% (−13 to −51%) HME: ΔFEV_1_ = −10% (0 to −26%)
Millqvist et al. (1995)	−10 Whole body exposure	Not reported	3 ×6	50–150 W	Study A, *n* = 9 No HME: ΔFEV_1_ = −36% HME: ΔFEV_1_ = −11% HME “protective effect” = 70% (range 19–100%) Two women could not complete protocol without HME Study B *n* = 5 HME “protective effect” = 74% Placebo mask with one-directional filters (no heat-moisture exchange), protective effect = 14%
Millqvist et al. (2000)	−10 Nasal clips to prevent nasal breathing	Not reported	3 ×6	Incremental 30–150 W	No HME: ΔFEV_1_ = −27% HME: ΔFEV_1_ = −12% Beta-2-agonist: ΔFEV_1_ = −7% HME+beta-2-agonist: ΔFEV_1_ = None
Jackson et al. (2018)^*^	8 Whole body exposure	20% RH	6	80% PPO	HME: ΔFEV_1_ = −6.0%, SHAM: ΔFEV_1_ = −9.5%, No HME: ΔFEV_1_ = −13.0%, *p* = 0.03 between HME and No HME.
Gravelyn et al. (1987)	22	0 mg H_2_O·L^−1^	5	60–70 L·min^−1^	No HME: 300% increase in sRaw after hyperpnea with dry air HME: 7% increase in sRaw after hyperpnea
Brenner et al. (1980)	23	26% RH	6	90% HR_*max*_	No HME: FEV_1_ = 66 ± 6 of individuals' (pre-exercise) baseline, 6 min after exercise HME: FEV_1_ = 91 ± 3% of individuals' baseline
Frischhut et al. (2020)	−20 Whole body exposure	46.2% RH	8	90–95% HR_*max*_	Winter athletes without asthma No HME: FVC −5.9%, FEV_1_ −4.2% vs. pre-exercise baseline HME: FVC no reduction FEV1 no reduction

*Conference proceedings.

*FEV_1_, Forced expiratory volume in 1 s; HME, Heat and moisture-exchanging breathing mask; HR, Heart rate; PPO, Peak power output; RH, Relative humidity; sRaw, Specific airways resistance; $\overset{\cdot }{V}$O_2_max, Maximum oxygen uptake; W, Watts*.

### What About HMEs for Healthy Athletes?

While the case to consider HMEs as preventative tools for exercise-induced asthma may be relatively well-supported by present data, we are aware that many athletes without asthma may also utilize HMEs. Given the higher risk of developing exercise-induced asthma among elite winter endurance athletes, investigation of the potential of HMEs to attenuate asthma-like symptoms, bronchial obstruction, or other biomarkers of airway injury is warranted. To our knowledge, only a single study to date has set out to evaluate potential prophylactic effects of HMEs to prevent airway damage or asthma in healthy athletes. Following a high-intensity exercise bout in −20°C, without use of an HME, Frischhut et al. (2020) reported post-exercise decreases in FVC and FEV_1_ in healthy athletes. HME usage attenuated these responses, and also resulted in fewer respiratory symptoms (Frischhut et al., 2020).

Anecdotal evidence suggests that HMEs are routinely used not only by individuals with asthma but also by otherwise healthy individuals training in very cold environmental conditions. It appears less common that healthy athletes utilize HMEs for high-intensity training sessions or in competition. The rationale underpinning this choice requires further investigation but could include improved comfort during training, reduction in respiratory symptoms and/or a belief that HMEs are protective for their airway health.

Given the higher risk of developing exercise-induced asthma among elite winter endurance athletes, investigation of the potential of HMEs to attenuate asthma-like symptoms, bronchial obstruction, or other biomarkers of airway injury is warranted. To our knowledge, no studies to date have evaluated potential prophylactic effects of HMEs to prevent airway damage or asthma in healthy athletes.

At present, a conservative approach may suggest that there is little risk for healthy athletes to use HMEs during training, but potentially high reward if HMEs are able to minimize airway injury and symptoms during exercise in very cold or dry environments. Nevertheless, avoidance of HME utilization by healthy athletes may occur for a multitude of reasons. Athletes may experience or perceive increased resistance to breathing and become concerned that this could impair their ability to perform in races, or complete high-intensity sessions as planned. Discomfort may occur if water vapor begins to freeze around the edges of the HME or around the filter area, and poor fit may become a distraction during training. Whilst a couple of studies suggest that healthy individuals can use a HME without discomfort or reduction in performance (Eiken et al., 1989; Seifert et al., 2017), it is likely that there is a large variation in comfort, fit and resistance to breathing among current, commercially-available HMEs. It is also possible that healthy athletes do not see a benefit to using HMEs, although, a recent study demonstrated that use of an HME during sprint exercise in −9°C attenuated the performance deficit seen without a HME in a cold environment; sprint performance in −9°C with a HME was similar to performance in room temperature (Seifert et al., 2017). Qualitative work could provide insight into current attitudes toward HME use in winter endurance athletes and inform manufacturers about whether current models are fit for purpose.

### When Should HMEs Be Used?

**Figure 3:**
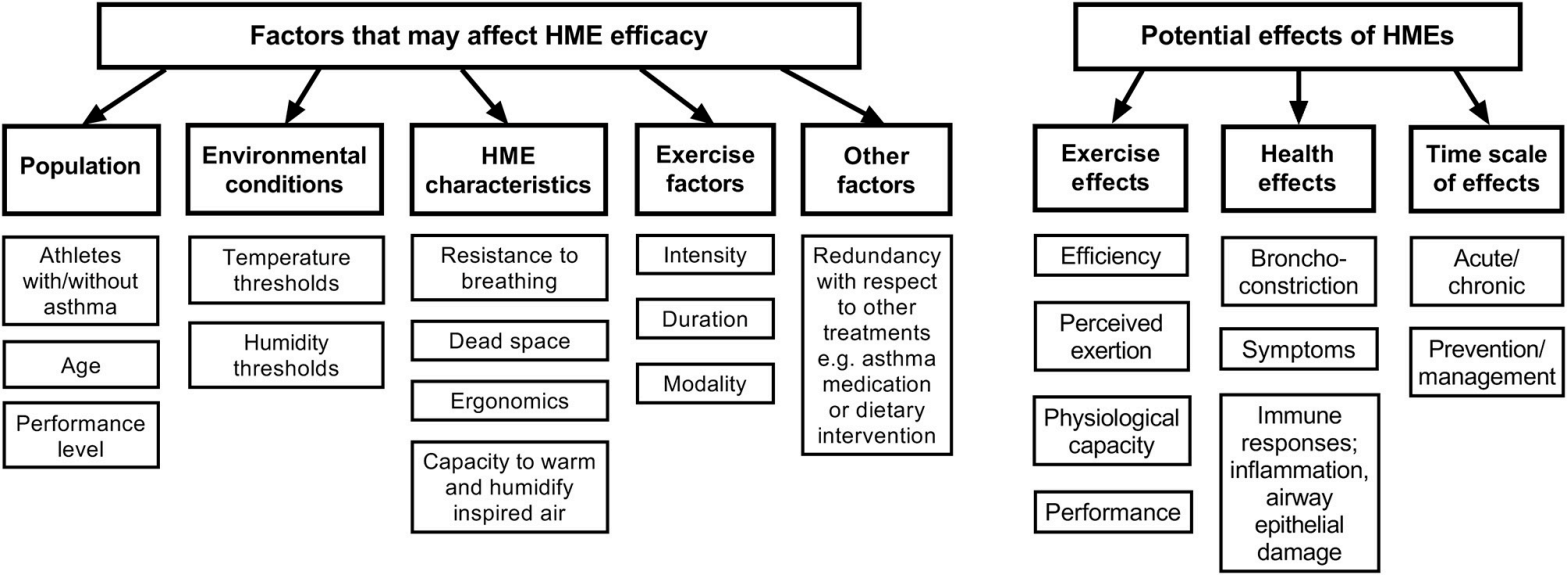
Conceptual model of potential effects and interactions of HME use by athletes and exercising populations.

With regard to recommendations for use of HMEs, many questions remain regarding temperature and/or humidity thresholds as well as exercise intensities at which use of HMEs may (or may not) be recommended for athletes with and without asthma. Numerous coaches and officials working in these sports have made a plea to us as sports physicians and scientists to recommend temperature and humidity thresholds below which risk of airway injury is substantially increased in junior and senior athletes, and further to provide information about whether HMEs are sufficient to counteract potential detrimental effects on the airways of healthy athletes during exercise in the cold. Table 1 summarizes the existing evidence for occurrence of airway responses at a range of sub-zero temperatures, and thus could be used to suggest thresholds below which use of HMEs might be appropriate for the healthy athlete, whereas Figure 3 outlines potential effects of HMEs as well as factors to consider that may affect HME efficacy.

To summarize, there is a lack of knowledge at present about the extent of positive and negative effects of HME use, precisely with respect to their capacity to heat and humidify inhaled air, the resistance they pose to breathing and effects they have on athletes' ventilation, energy cost and performance. Given their observed effects on FEV_1_ after exercise challenge, current data suggests HMEs have potential to mitigate exercise-induced asthma. If performance capacity via discomfort, distraction or breathing resistance remains a concern, utilization of an HME during the warm-up only could be worth considering, since warm-up may be beneficial in preventing subsequent EIB (Stickland et al., 2012); and because rapid transition from warm to cold environments (and back again) may provoke reactive hyperemia (Gilbert and McFadden, 1992). However, due to a paucity of data, such strategies would require further investigation. Future work should also examine the potential of HMEs to attenuate airway damage and inflammation as well as respiratory symptoms both in subjects with asthma and healthy individuals undertaking exercise training in sub-zero environments.

## Conclusions

It is well-understood that exercise in cold and dry air can trigger airway inflammation and epithelial injury and may be associated with increased prevalence of airway hyper-responsiveness and asthma. Whilst this relationship has been understood for several decades, the prevalence of asthma in senior cross-country skiers in Scandinavia has not changed in this time, and so investigation of preventative strategies is warranted.

Current understanding suggests that repeated airway injury leads to development of inflammation and airway hyper-responsiveness, and that this process is exacerbated by cold and dry air. While FEV_1_ is commonly used as a primary endpoint for studies in athletes with asthma, primary endpoints for the effect of cold air on the airways of healthy athletes have not been determined. Current temperature limits for competition in winter endurance sports make no specific reference to effects of the environment on the airways. A small body of evidence suggests that HMEs may attenuate exercise-induced bronchoconstriction, but existing data are discordant regarding environmental conditions where exercise may be harmful to the airways in healthy athletes or athletes who have already developed airway hyper-responsiveness. Further work is required to characterize temperature and humidity thresholds as well as exercise intensities and durations for which HME use may be recommended. Based on the limited evidence currently available, we suggest that for athletes with asthma, use of HMEs during training in sub-zero temperatures or dry environments could be a low-risk and high-return strategy to protect the airways. Athletes with healthy airways may be encouraged to use HMEs at sub-zero temperatures where airway discomfort arises, as a low-risk and *potential* prevention strategy against airway damage. However, further work is required to investigate the prophylactic potential of HMEs for both short-term attenuation of airway damage and respiratory symptoms in healthy athletes, as well as long-term prevention of asthma in winter endurance athletes.

## Author Contributions

HH, MA, and NS conceived the idea for the manuscript, drafted the manuscript, provided critical review, and approved the final version for publication.

## Conflict of Interest

In May 2019 the authors were provided with 4 complimentary HMEs with disposable filters for use in forthcoming research by Vapro Produktutveckling AB, Västerås, Sweden.
